# The transcription factor *GhWRKY70* from *gossypium hirsutum* enhances resistance to verticillium wilt via the jasmonic acid pathway

**DOI:** 10.1186/s12870-023-04141-x

**Published:** 2023-03-14

**Authors:** Shuling Zhang, Lijun Dong, Xue Zhang, Xiaohong Fu, Lin Zhao, Lizhu Wu, Xingfen Wang, Jianfeng Liu

**Affiliations:** 1 School of Life Sciences, Institute of Life Science and Green Development, Hebei University, No.180, Wusi East Road, 071000 Baoding, China; 2 State Key Laboratory of North China Crop Improvement and Regulation, Hebei Agricultural University, Baoding, China

**Keywords:** Cotton, Transcription factor, GhWRKY70, Verticillium wilt, Jasmonic acid pathway

## Abstract

**Background:**

The WRKY transcription factors play significant roles in plant growth, development, and defense responses. However, in cotton, the molecular mechanism of most WRKY proteins and their involvement in Verticillium wilt tolerance are not well understood.

**Results:**

*GhWRKY70* is greatly up-regulated in cotton by *Verticillium dahliae*. Subcellular localization suggests that GhWRKY70 is only located in the nucleus. Transcriptional activation of GhWRKY70 further demonstrates that GhWRKY70 function as a transcriptional activator. Transgenic *Arabidopsis* plants overexpressing *GhWRKY70* exhibited better growth performance and higher lignin content, antioxidant enzyme activities and jasmonic acid (JA) levels than wild-type plants after infection with *V. dahliae*. In addition, the transgenic *Arabidopsis* resulted in an enhanced expression level of *AtAOS1*, a gene related to JA synthesis, further leading to a higher JA accumulation compared to the wild type. However, the disease index (DI) values of the VIGS-treated cotton plants with *TRV:WRKY70* were also significantly higher than those of the VIGS-treated cotton plants with *TRV:00*. The chlorophyll and lignin contents of *TRV:WRKY70* plants were significantly lower than those of *TRV:00* plants. *GhAOS1* expression and JA abundance in *TRV:WRKY70* plants were decreased. The GhWRKY70 protein was confirmed to bind to the W-box element in the promoter region of *GhAOS* by yeast one-hybrid assay and transient expression.

**Conclusion:**

These results indicate that the GhWRKY70 transcription factor is a positive regulator in Verticillium wilt tolerance of cotton, and may promote the production of JA via regulation of *GhAOS1* expression.

**Supplementary Information:**

The online version contains supplementary material available at 10.1186/s12870-023-04141-x.

## Background

Cotton (*Gossypium hirsutum* L.) is an excellent source of oil and fiber and is widely considered one of the most economically important crops worldwide. In the growth and development process, cotton is highly vulnerable to attack by *V. dahliae*, which is a typical soil borne pathogenic fungus that results in Verticillium wilt disease in more than 400 dicotyledon plant species, including annual herbs, perennials, and woody plants [1]. The yield of cotton declined by 10–35% because of Verticillium wilt. Production practices have demonstrated that genetic engineering by expressing important disease resistance genes is an effective approach for developing transgenic plants with enhanced disease resistance [2, 3]. Therefore, it is an urgent task to understand the underlying physiology and molecular mechanism in cotton defense against *V. dahliae* infection.

A large spectrum of resistance related genes were regulated to participate in the synthesis of various metabolites that resist pathogen infection in plants. JA plays an important role in diverse physiological and developmental processes and biological and abiotic stresses [4–7]. In the JA signal transduction pathway, jasmonate ZIM domain (JAZ) protein is a negative regulator and JA induces its degradation to activate JA responsive gene expression [8–10]. *MYC2*, one of the master transcription factors in the JA signaling pathway, is the target gene of JAZ proteins which operate in diverse JA-dependent functions and various plant environmental responses [11–13]. As a stress signaling compound, the rapid accumulation of JA cannot be fulfilled without lipoxygenase (LOX) and allene oxides synthase (AOS), two key enzymes in the synthesis of JA [14]. RNAi-mediated silencing of *GhLOX2* decreased cotton resistance to *V. dahliae* and was coupled with suppression of JA-related genes both after inoculation with *V. dahliae* and jasmonic acid methyl ester (MeJA) treatment [15]. The *AOS* mutant of *Arabidopsis thaliana* was susceptible to *Sclerotinia sclerotiorum* accompanied by deficiency of JA biosynthesis [16]. Although *LOX2* and *AOS* may contribute to responses to biological stresses by regulating JA levels, the gene expression mechanism remains unclear.

Plants challenged by pathogen infection can produce reactive oxygen species (ROS), which can induce plants to activate some stress-related genes to resist environmental changes [17, 18]. Hydrogen peroxide (H_2_O_2_), as a more physiologically important ROS, is a relatively stable ROS that can diffuse into subcellular compartments. H_2_O_2_ can be generated and cleared by many enzymes, such as peroxidase (POD), superoxide dismutase (SOD) and catalase (CAT), which are involved in the antioxidant system. The role of H_2_O_2_ in the extracellular matrix is not limited to participating in a defense response but is also involved in regulating the synthesis of cell wall components, such as lignin synthesis [19]. Overaccumulation of H_2_O_2_ can damage plant biological macromolecules, disturbing normal physiological and metabolic processes in plant cells [20–23]. Therefore, plants adjust the concentration of H_2_O_2_in vivo by regulating the activities of antioxidant enzymes to resist pathogen invasion.

WRKYs, as transcription factors (TFs), are important constituents of plant signaling pathways and play crucial roles in controlling many important biological processes [24, 25]. Based on the number of WRKY domains and the structure of their zinc-finger motifs, WRKY proteins can be classified into three main groups: I, II, and III. Group I contains two WRKY domains and a C_2_H_2_ (CX_4 − 5_CX_22 − 23_HXH) zinc finger. One WRKY domain and a C_2_H_2_ motif or a C_2_HC (CX_7_CX_23_HXC) motif exist in Group II a-e and Group III, respectively [26]. Group III is notably different from Groups I and II, is expressed in ferns and some eukaryotic cells as well as higher plants and is expressed only in higher plants, in which most expressed proteins are related to biological stress [27]. Therefore, Group III TFs may have evolved as a result to acquire adaptations to different environmental pressures [28, 29]. *AtWRKY70* (Group III) is a node of convergence for salicylic acid (SA)- and JA-mediated defense signaling pathways. Together with WRKY54 (Group III), *AtWRKY70* enhances resistance to the hemibiotroph *Pseudomonas syringae* pv tomato (Pst) DC3000 but increases susceptibility to the necrotrophic pathogens *Pectobacterium carotovorum* and *Botrytis cinerea* in *Arabidopsis* [30]. Although the identification and characterization of the WRKY gene family have been performed in *Gossypium arboreum*, *Gossypium raimondii*, and *G. hirsutum* [31, 32], only a few WRKY family genes resistant to *V. dahliae* have been identified in cotton, such as *GbWRKY1* [33] and *GhWRKY70* [34, 35]. The molecular mechanisms of cotton resistance to *V. dahliae* invasion are unclear.

There are many studies related to biotic and abiotic stress of the WRKY transcription factor family. Cotton is considered one of the most economically important crops worldwide. It is of great significance to analyze the molecular mechanism of Verticillium wilt tolerance in cotton. However, the molecular mechanism of WRKY genes responding to Verticillium wilt tolerance remains unclear in cotton. Here, we identified a new WRKY transcription factor, *GhWRKY70*, which can regulate *GhAOS1* expression by directly binding to the w-box in the *GhAOS1* promoter. Furthermore, our study indicated that *GhWRKY70* plays a positive regulatory role in the response to invasion of *V. dahliae*, which was at least in part through increasing the content of JA by promoting *GhAOS1* expression.

## Results

### Isolation and bioinformatics analysis of *GhWRKY70*

**Fig. 1:**
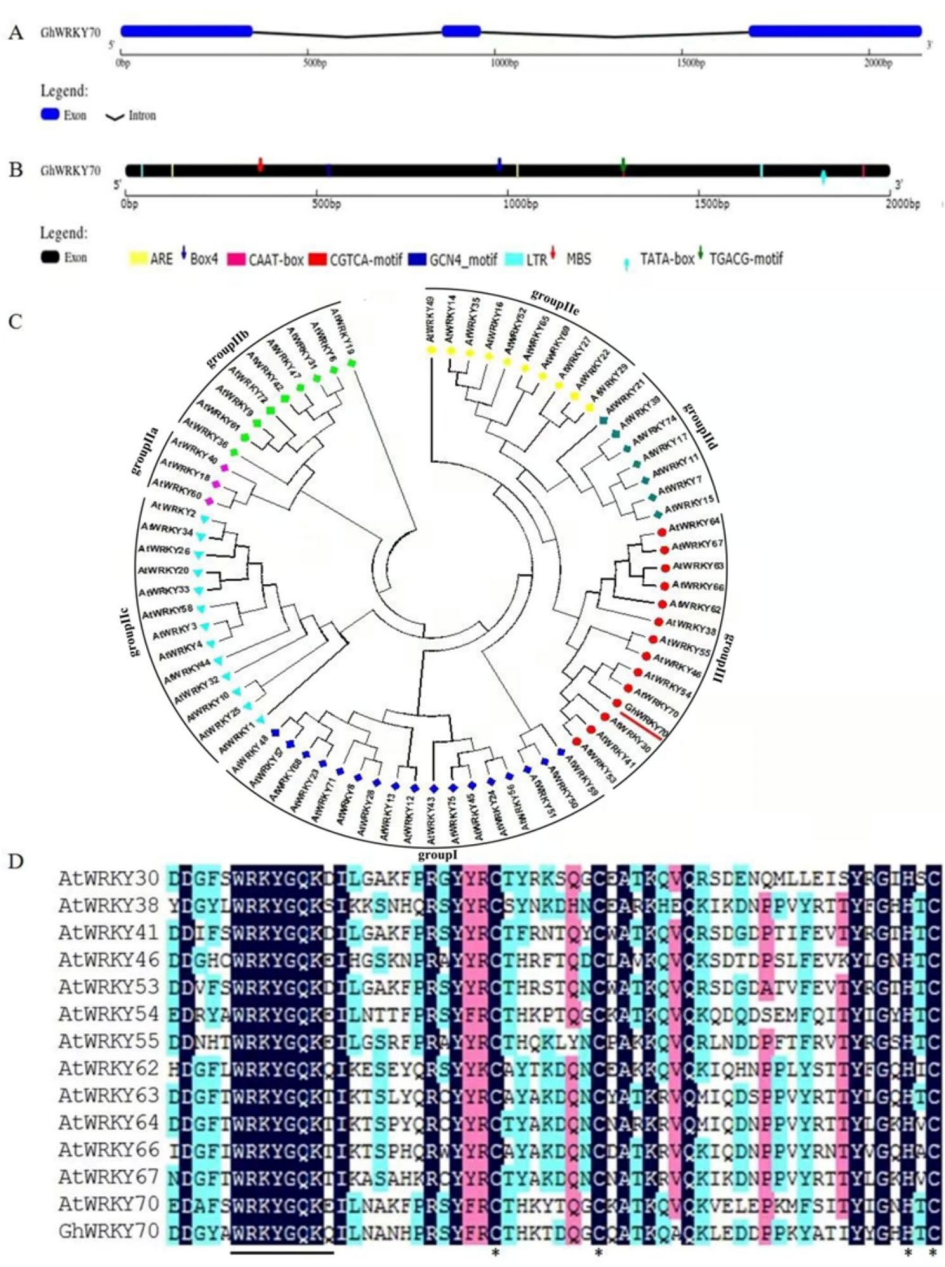
Sequence analysis of GhWRKY70. (**A**) Schematic diagram of the *GhWRKY70* structure. (**B**) Cis-element analysis of *GhWRKY70* in the promoter region. (**C**) Phylogenetic tree constructed by WRKYs of *Arabidopsis thaliana* and GhWRKY70. (**D**) Multiple alignments of WRKY domains between *Arabidopsis thaliana* Group III WRKYs and GhWRKY70. The blue background shows identical amino acids. The conserved WRKY motif is represented by a line while the zinc-finger structures are shown using asterisks

A significantly induced WRKY TF named *GhWRKY70* was obtained from a transcriptome of *G. hirsutum* following *V. dahliae* infection. Bioinformatics analysis showed that the length of *GhWRKY70* was 2141 bp. *GhWRKY70* comprises a section of spliced DNA containing three exons and two introns. The sizes of the introns were 505 and 715 bp, respectively (Fig. 1A). To determine the cis-acting elements, 2.0 kb promoter regions of DNA sequences upstream from the codons of *GhWRKY70*, were identified and analyzed by plant CARE (Fig. 1B). Some basic elements, including TATA elements and CAAT-boxes, were found. In addition, cis-elements related to abiotic stress and hormone regulation were also identified in the promoter region. Examples include LTR (cis-acting element involved in low-temperature responsiveness), MBS (MYB binding site involved in drought-inducibility), CGTCA-motif and TGACG element (cis-acting element involved in the MeJA response). *GhWRKY70* contains a 921 bp open reading frame (ORF) and encodes a predicted polypeptide of 306 amino acid residues with a calculated molecular mass of 34.3 kDa and an isoelectric point of 6.06. A phylogenetic tree was constructed based on GhWRKY70 using a total of 72 WRKYs from *Arabidopsis* (Table S1) and demonstrated that they could be classified into four major groups; GhWRKY70 had the closest relationship with AtWRKY70 due to its high identity and belonged to the Group III (Fig. 1C). The gene was designated as GhWRKY70. Multiple sequence alignment showed that the GhWRKY70 protein possesses a highly conserved WRKY domain comprising 60 amino acids (133–192) (Fig. 1D).

### Expression patterns of *GhWRKY70* in response to abiotic and biotic stresses

**Fig. 2:**
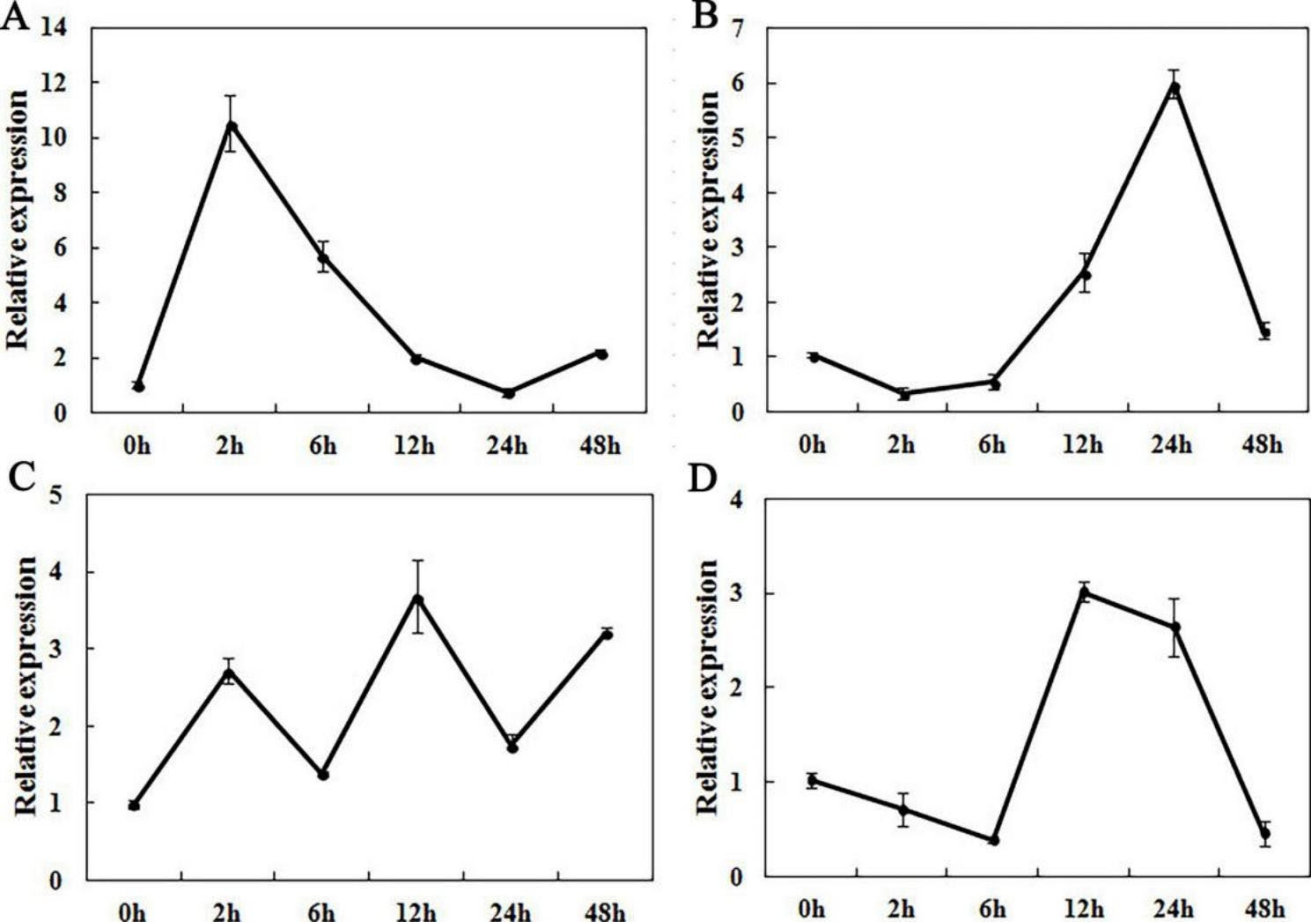
**E**xpression patterns of *GhWRKY70*. Expression levels of *GhWRKY70* under different treatments with *V. dahliae*. (**A**), MeJA (**B**), PEG (**C**) and low temperature (**D**). For each treatment, the expression level at 0 h was set as 1.0 and the data represent the means ± SE of three replicates

According to the cis-elements of *GhWRKY70* in the promoter region, the expression of *GhWRKY70* under different abiotic and biotic stresses, including *V. dahliae*, MeJA, polyethylene glycol (PEG–6000) and low temperature, was investigated by qRT‒PCR. The results showed that the *GhWRKY70* transcript level quickly accumulated at 2 h after infection with *V. dahliae*. (higher than tenfold induction), decreased to a low value at the last time point until 24 h, and then increased at 48 h (Fig. 2A). After MeJA treatment, the transcript abundance of *GhWRKY70* first decreased and then increased to the maximum value at 24 h (Fig. 2B). In the case of PEG treatment, the expression level of *GhWRKY70* was slightly up-regulated (Fig. 2C). When subjected to cold treatment, the expression of *GhWRKY70* eventually declined at 48 h, probably because the mRNA degraded gradually after long-term cold treatment of plants (Fig. 2D).

### Subcellular localization of GhWRKY70

**Fig. 3:**
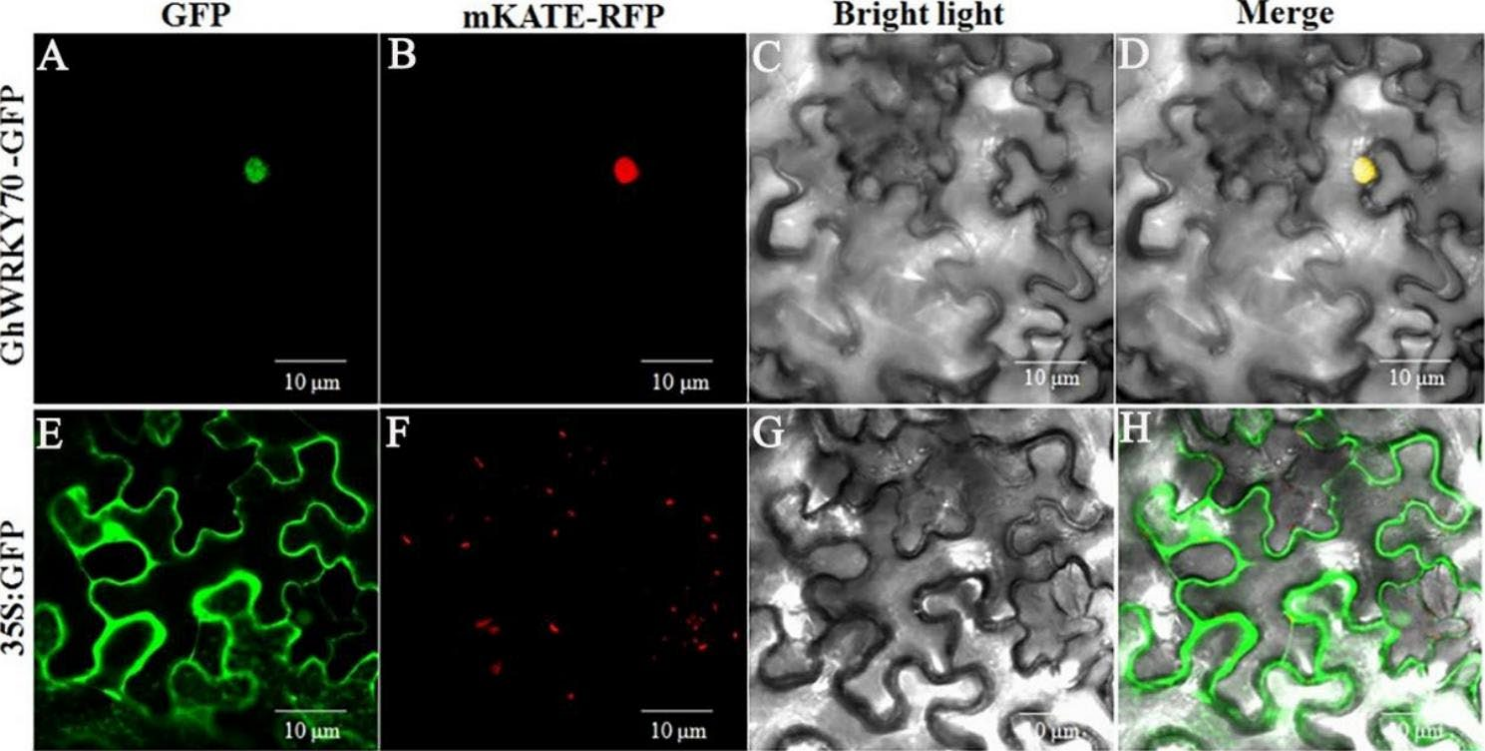
Subcellular localization of GhWRKY70. (**A** and **B**) Tobacco epidermal cells were transformed with constructs containing the fusion plasmids GhWRKY70::GFP and 35 S::nls::mKate::RFP. (**C** and **D**) Images under bright field and merge. (**E** and **F**) Tobacco epidermal cells were transformed with a construct containing the pSuper1300-GFP vector and 35 S::nls::mKate::RFP. (**G** and **H**) Images under bright field and merge. mKATE-RFP as a nuclear marker. Scale bar = 10 μm

Subcellular localization of GhWRKY70 was predicted by examining GhWRKY70::GFP. The nuclear signal peptide fused to the RFP protein mKate was used as a positive control. The GhWRKY70::GFP vector and positive control with red fluorescent protein (RFP)-mKATE were cotransformed into *Nicotiana tabacum* leaves. The green fluorescence from GhWRKY70::GFP perfectly overlapped with the red fluorescence from (RFP)-mKATE and was exclusively detected under a confocal microscope (Fig. 3A–D). However, green fluorescence was observed in the entire cell region when only the GFP plasmid and red fluorescent protein (RFP)-mKATE vector were cotransformed into tobacco leaves. The results indicated that GhWRKY70 was localized in the nucleus (Fig. 3E–H).

### Transcriptional activation assay of GhWRKY70 in yeast

**Fig. 4:**
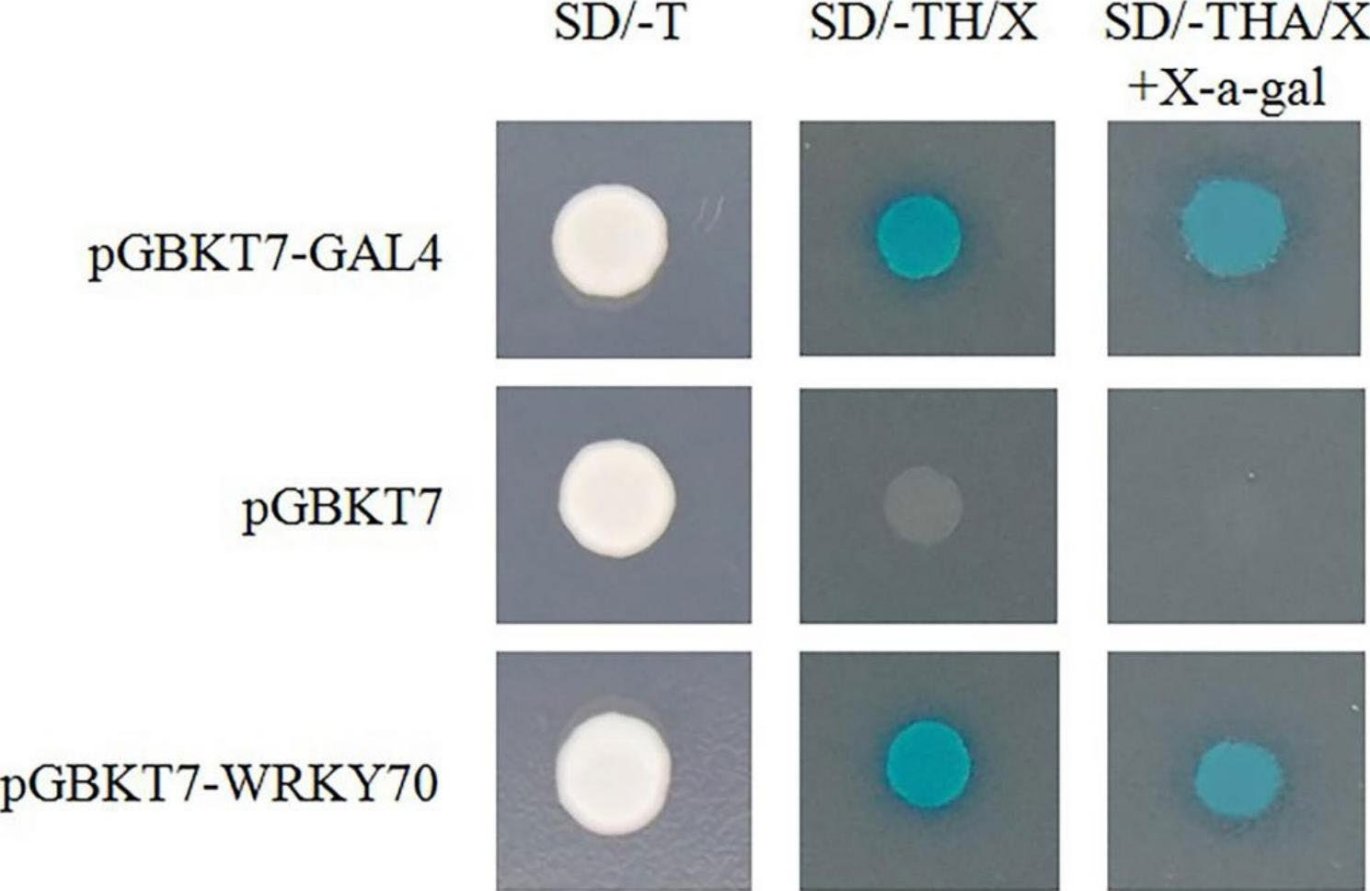
Transcriptional activity assay of GhWRKY70 in yeast. The transformed yeast cells were cultured on SD-T, SD-TH/X, and SD-THA/X with X-α-gal medium

The GAL4 yeast expression system was used to detect the transcriptional activation of GhWRKY70. Yeast strain AH109 was transformed with the constructs pGBKT7-GhWRKY70 and pGBKT7-GAL4 as positive controls and pGBKT7 as a negative control. The yeast cells transformed with pGBKT7-GhWRKY70 and pGBKT7-GAL4 grew well and turned blue on SD-THA/X medium with X-α-gal. Meanwhile, the yeast cells transformed with pGBKT7 could only exist on the SD/-TH/X medium (Fig. 4, Fig. S1). The results demonstrated that GhWRKY70 functioned as a transcriptional activator.

### Overexpression of *GhWRKY70* improved *Arabidopsis* resistance to *V. dahliae*

**Fig. 5:**
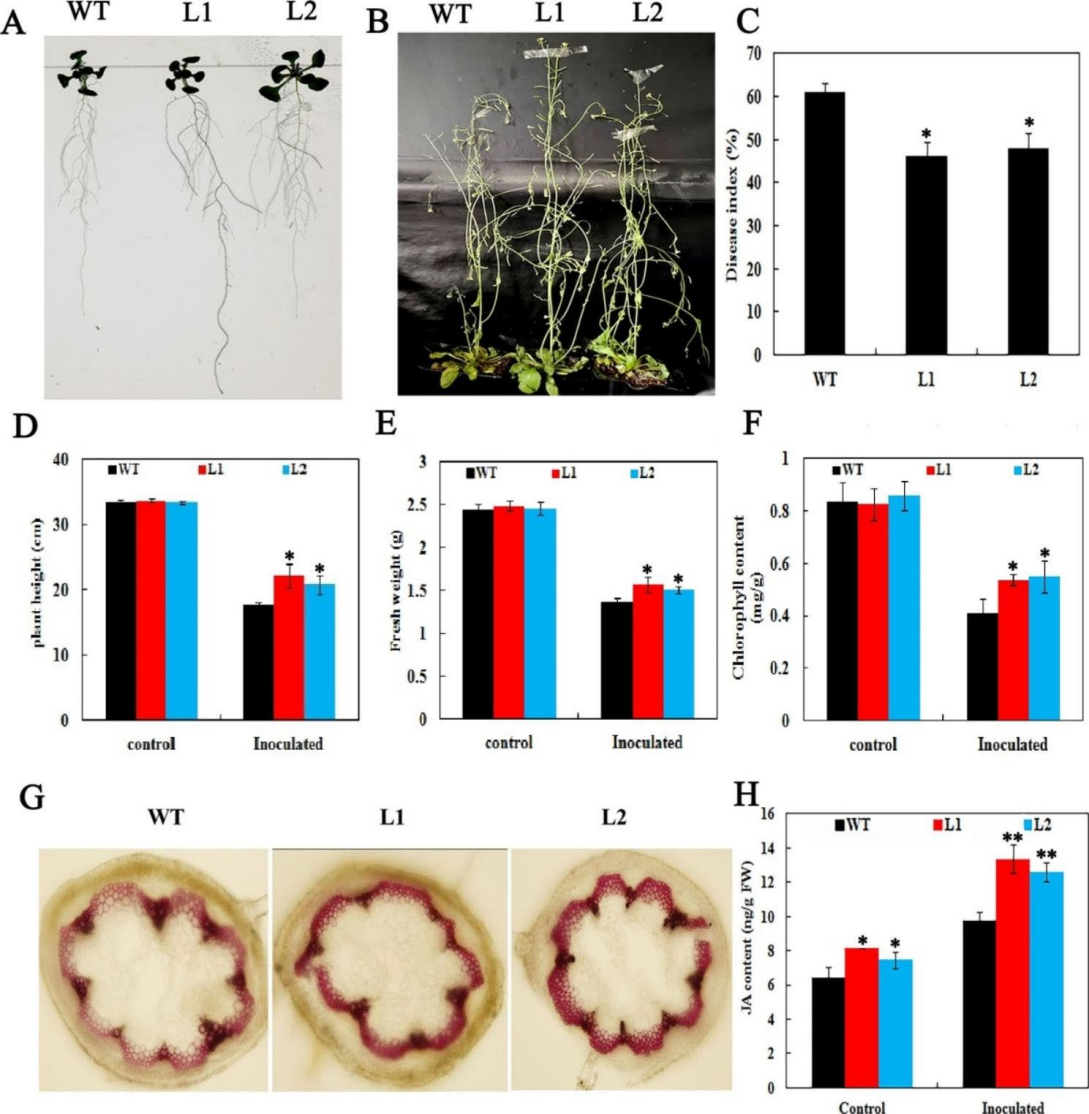
Resistance identification of transgenic *Arabidopsis* to *V. dahliae*. (**A**) Root phenotypes of transgenic and WT *Arabidopsis* (9 days). (**B**) Phenotypes of transgenic lines and WT plants after inoculation with spore suspensions (1.4 × 10^7^ conidia mL^− 1^) for 15 days. (**C**) DI values for WT and transgenic *Arabidopsis*. (**D**, **E** and **F**) The plant height, fresh weight and chlorophyll content for WT and transgenic *Arabidopsis*. (**G**) Stem staining of WT and transgenic *Arabidopsis* after *V. dahliae* infection (15 days). (**H**) The content of JA in the WT and transgenic *Arabidopsis* after *V. dahliae* infection (15 days)

*GhWRKY70* transcript levels were strongly induced by *V. dahliae* and MeJA, whereas transgenic *Arabidopsis* plants overexpressing *GhWRKY70* were generated to characterize the role of *GhWRKY70* in response to *V. dahliae*. Two transgenic lines with higher transcriptional levels of *GhWRKY70* (named L1 and L2) were selected for the resistance experiment. Nine days after germination, the roots of the transgenic lines were longer and stronger than those of the wild type (WT) (Fig. 5A, Fig. S2A). To assess the effect of damage caused by *V. dahliae*, the four-week-old WT and transgenic plants were inoculated with spore suspensions of *V. dahliae*, and the control was treated with water instead of *V. dahliae*. After withholding and culturing for 15 days, transgenic plants displayed better resistance than the WT plants (Fig. 5B). The disease index (DI), is a comprehensive index for measuring the incidence rate and severity of plant disease. In our study, the DI was approximately 46.1% and 48.3% in the two transgenic lines, which was lower than the 61.0% DI of the wild type (Fig. 5C), suggesting that Verticillium wilt damage in the transgenic *GhWRKY70* lines was milder than that in WT *Arabidopsis*. The height and fresh weights of WT inoculated plants were 46.6% and 41.8% lower than the mock control, respectively. The height and fresh weights from the two inoculated transgenic L1 and L2, by contrast, were 34.4% and 37.6% and 36.5% and 38.5% lower than those of the control, respectively (Fig. 5D, E). Similarly, the chlorophyll content of the transgenic lines was remarkably higher than that of the WT (Fig. 5F). Lignin staining showed that the lignin content of the transgenic lines increased (Fig. 5G). Taken, our results showed that transgenic *Arabidopsis* had obviously increased *V. dahliae* resistance. Moreover, there is a JA-inducing element in the promoter sequence of *GhWRKY70*, and the expression level of *GhWRKY70* was induced by MeJA. Therefore, JA levels of the WT and transgenic lines were quantitatively examined in this study. As shown in Fig. 5H, JA levels of the transgenic L1 and L2 increased by 26.8% and 16.2% compared with the WT, respectively. JA levels of the WT and transgenic lines all increased, while the increase of JA content in transgenic lines was significantly higher than that of WT plants under *V. dahliae* induction.

### Silencing of *GhWRKY70* in cotton confers sensitivity to *V. dahliae*

**Fig. 6:**
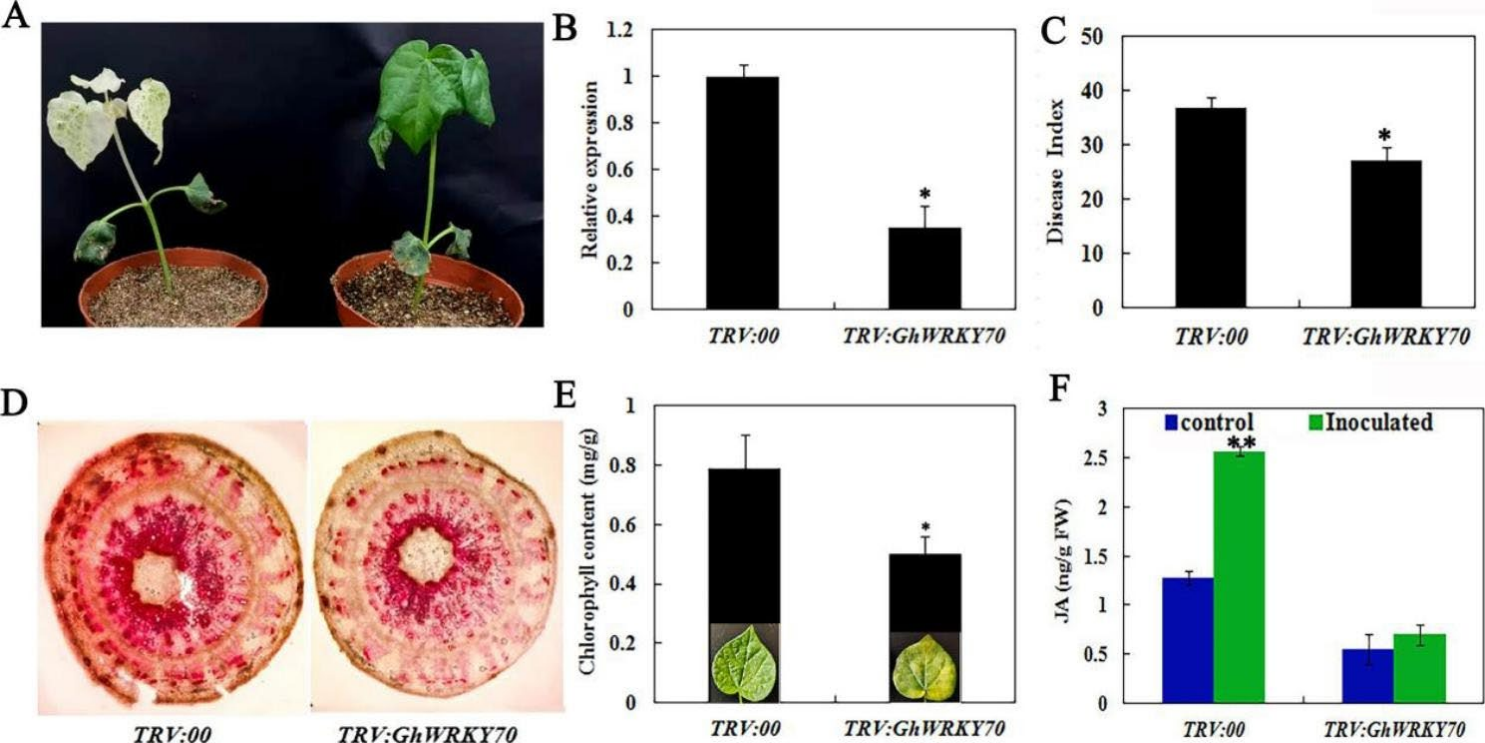
VIGS of *GhWRKY70* in cotton. (**A**) *TRV:00* (right) and *TRV:GhCLA1* (left ) seedlings of cotton (**B**) Relative expression of *GhWRKY70* in empty-vector control (*TRV:00*) and *GhWRKY70*-silenced (*TRV:GhWRKY70*) cotton plants.(**C**) DI values of *TRV:GhWRKY70* and *TRV:00* plants after *V. dahliae* infection. (**D**) Staining of lignin in *TRV:GhWRKY70* and *TRV:00* plants stem after *V. dahliae* inoculation (15 days). (**E** and **F**) The content of chlorophyll and JA in *TRV:GhWRKY70* and *TRV:00* plants

To further elucidate the role of *GhWRKY70* in resistance to *V. dahliae*, *GhWRKY70*-silenced plants were obtained by a virus-induced gene silencing (VIGS) method. When the *TRV:GhCLA1* plants showed a leaf bleaching phenotype (Fig. 6A), the transcripts of *GhWRKY70* were analyzed in the silenced plants and *TRV:00* plants. The results showed that the expression of *GhWRKY70* was reduced in the *TRV:GhWRKY70* plants compared to the *TRV:00* plants (Fig. 6B). When subjected to *V. dahliae* treatment for 15 days, the proportion of DI values was investigated and showed that the silenced plants were significantly higher than those of the *TRV:00* plants (Fig. 6C, Fig. S2B). Moreover, the lignin content and chlorophyll content of the silenced plants were significantly lower than those of the *TRV:00* plants (Fig. 6D and E). In addition, JA levels of *TRV:00* plants increased by 50.1% after inoculation with *V. dahliae*, while *TRV:WRKY70* plants increased by only 29.6% (Fig. 6F).

### Silencing *GhWRKY70* affected antioxidant and defense enzymes of transformed plants

**Fig. 7:**
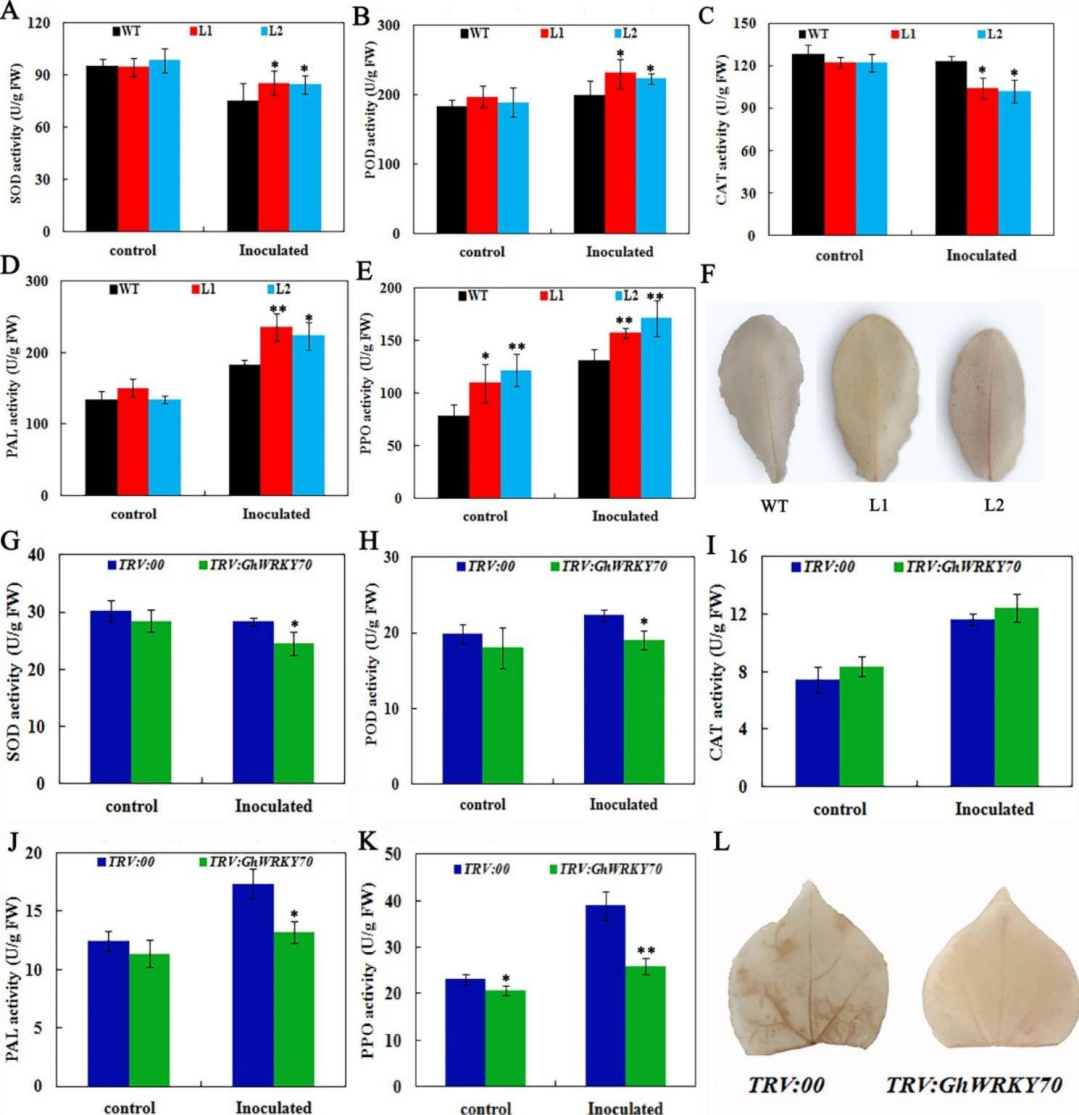
Analysis of antioxidant and defense enzyme activities and DAB staining in *Arabidopsis* and cotton treated with *V. dahliae* for 15 days. Activity of SOD (**A**), POD (**B**), CAT (**C**), PAL (**D**) and PPO (**E**) in transgenic and WT *Arabidopsis* treated with water or *V. dahliae*. DAB staining in WT and transgenic *Arabidopsis* leaves after *V. dahliae* infection (**F**). Activity of SOD (**G**), POD (**H**), CAT (**I**), PAL (**J**) and PPO (**K**) in *TRV:GhWRKY70* and *TRV:00* plants treated with water or *V. dahliae*. DAB staining in *TRV:GhWRKY70* and *TRV:00* leaves after *V. dahliae* infection (**L**)

To assess the changes in the antioxidant defense system created by overexpression and silencing *GhWRKY70*, the activities of antioxidant and defense enzymes in transgenic *Arabidopsis* lines (L1 and L2) and silenced cotton plants were measured and the results are shown in Fig. 7. The activities of antioxidant and defense enzymes in the two transgenic lines exhibited the same levels in comparison to the WT plants except for PPO. The SOD and POD activities also increased by 28.7% and 19.7% in L1 and 25.7% and 15.7% in L2, respectively, after 15 days of inoculation in comparison to the WT plants (Fig. 7A and B). The CAT activities in L1 and 2 were 15.4% and 17.1% lower than those in WT plants, respectively (Fig. 7C). Similarly, phenylalanine ammonialyase (PAL) and polyphenol oxidase (PPO) in transgenic L1 and L2 plants were 28.9% and 19.7% and 22.9% and 30.6% higher than those in the WT plants, respectively (Fig. 7D and E). Meanwhile, the DAB staining assay showed that the accumulation of H_2_O_2_ in both transgenic lines increased after 15 days of inoculation (Fig. 7F). The activities of PPO in *TRV:GhWRKY70* plants were lower than those in the *TRV:00* plants, while the activities of other antioxidant and defense enzymes showed no obvious differences between *TRV:GhWRKY70* and *TRV:00* plants. Fifteen days after inoculation, the SOD and POD activities in *TRV:GhWRKY70* were 13.4% and 14.8% lower than those in control plants, respectively (Fig. 7G and H), whereas the CAT activity was 6.57% higher than that in *TRV:00* plants (Fig. 7I). The PAL and PPO activities decreased 23.8% and 34.6% in *TRV:GhWRKY70* compared with *TRV:00* plants (Fig. 7J and K). In addition, the accumulation of H_2_O_2_ in *TRV:GhWRKY70* plants decreased more than that in *TRV:00* plants after 15 days of inoculation (Fig. 7L).

### Expression analysis of stress-responsive genes of transformed plants

**Fig. 8:**
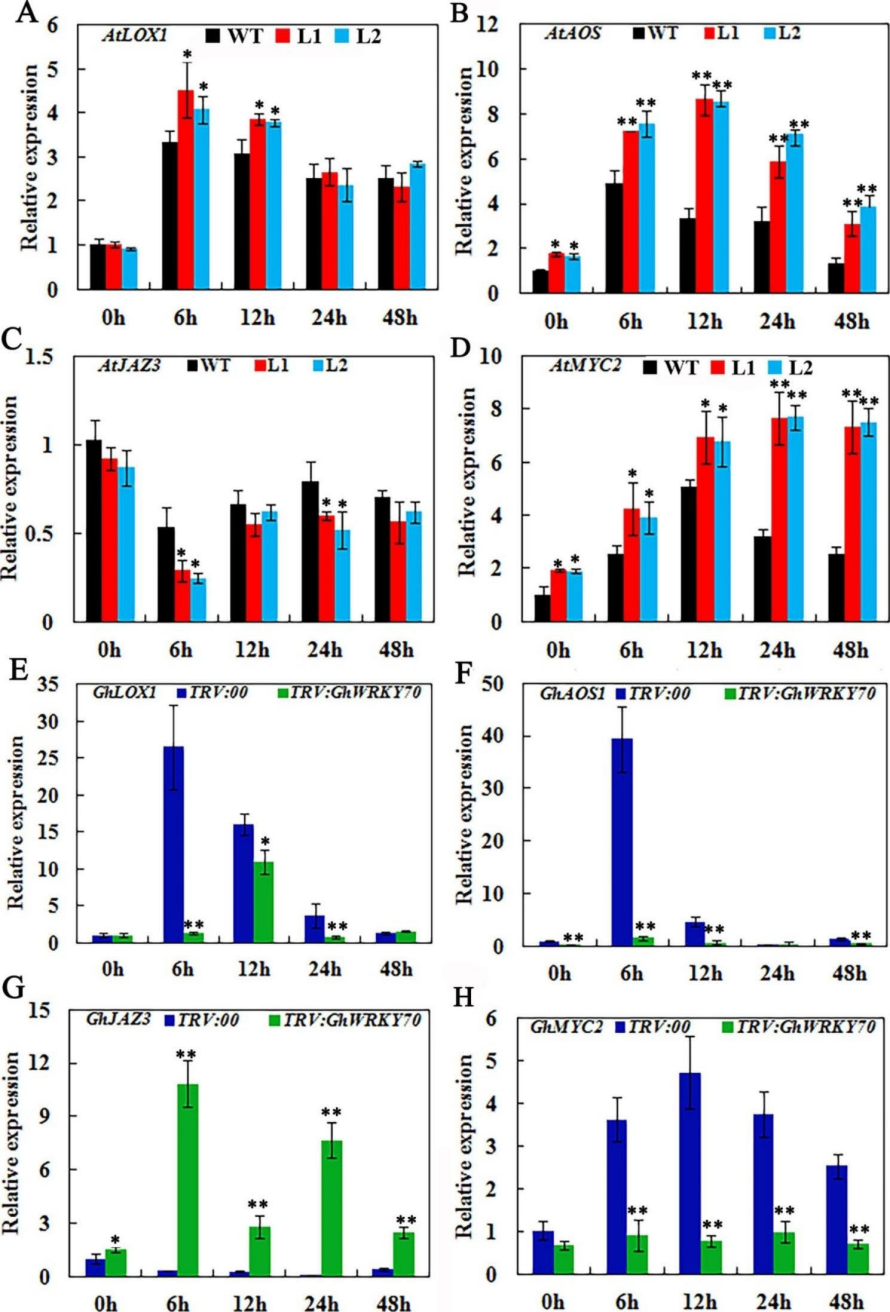
Expression profiles of the four JA signaling pathway genes in *Arabidopsis* (**A**, **B**, **C** and **D**) and cotton (**E**, **F**, **G** and **H**) before and after infection with *V. dahliae*

Our above data showed that the JA content in overexpression lines and silenced cotton plants significantly changed after *V. dahliae* treatment. Therefore, the genes related to the JA signaling pathway in the transformed plants were analyzed by qRT‒PCR. The expression of all genes in the transgenic lines and silenced plants was similar to that in the control plants under normal conditions. However, *AtLOX1* (AT1G55020) and *AtAOS* (AT5G42650) (for JA biosynthesis) in both transgenic lines had more abundant expression levels after *V. dahliae* treatment (Fig. 8A and B). For the JA signal response gene, the level of *AtJAZ3* (AT3G17860) gene expression was reduced, whereas *AtMYC2* (AT1G32640) expression was increased (Fig. 8C and D). Interestingly, *GhLOX1* (XM_041108768.1), *GhAOS1* (XM_016842008.2) and *GhMYC2* (XM_016865820.2) were significantly inhibited in *TRV:GhWRKY70* plants after *V. dahliae* treatment (Fig. 8E, F and H), whereas the expression of *GhJAZ3* (XM_041107142.1) increased significantly (Fig. 8G). The results perfectly matched the JA levels in the transgenic lines and silenced plants (Figs. 5H and 6F). Meanwhile, *V. dahliae* induction caused dramatic up-regulation of *GhLOX1* and *GhAOS1* in the *TRV:00* plants, but there were no significant changes in the *TRV:GhWRKY70* plants. All results suggested that *GhWRKY70* functions in response to *V. dahliae* by enhancing the expression of JA biosynthesis genes, especially *GhLOX1* or *GhAOS1*.

### GhWRKY70 directly interacts with the promoter of *GhAOS1*

**Fig. 9:**
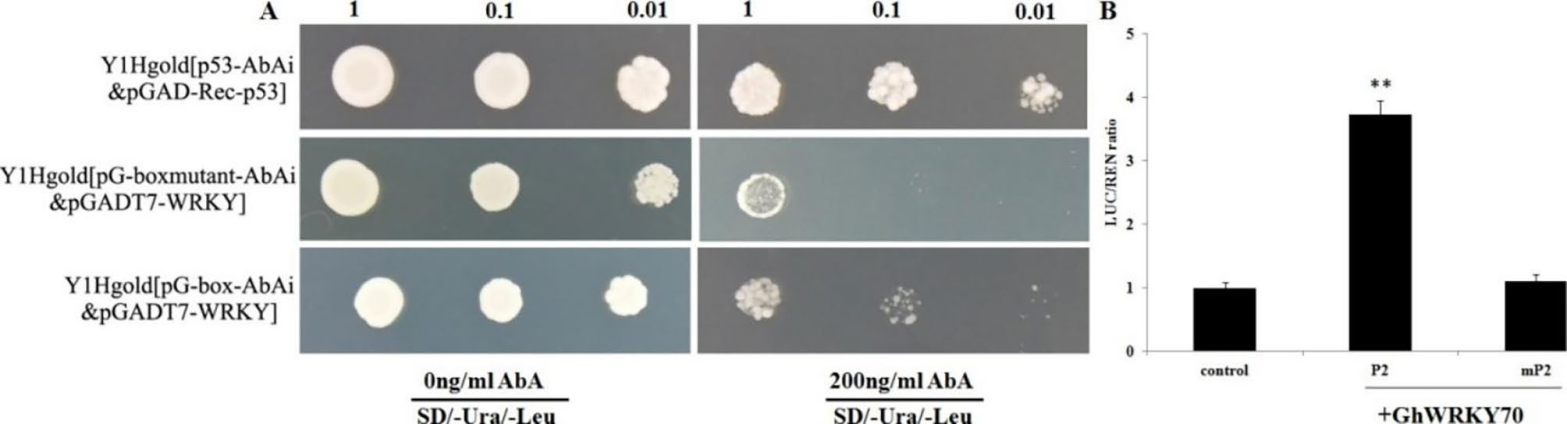
Interaction detection between GhWRKY70 and the *GhAOS1* promoter. (**A**) The yeast one-hybrid assay. (**B**) Transient expression assay of promoter activity

The expression levels of *GhAOS1* were strongly induced in *GhWRKY70*-overexpressing lines and decreased in *GhWRKY70*-silenced lines. We propose that *GhAOS1* might be a potential target gene regulated by GhWRKY70. The results of *GhAOS1* promoter sequence bioinformatics analysis showed that one potential W-box (TTGACT) element existed in the upstream region. To investigate the interaction between GhWRKY70 and the *GhAOS1* promoter, a yeast one-hybrid (Y1H) assay was conducted. The results showed that the yeast cells of positive controls and those cotransformed pGADT7-GhWRKY70 with a bait 150 bp fragment containing the W-box prey or W-box mutant (negative controls) grew normally in SD/-Ura/-Leu medium. However, when 200 ng/mL AbA was added to SD/-Ura/-Leu medium, the growth of the positive control and bait‒prey transformants survived, while the negative control was completely inhibited (Fig. 9A, Fig S3). Transient expression assays showed that the LUC/REN ratio in the tobacco leaf transformed with the P2 containing reporter and effector was significantly higher than that transformed with mP2 and effector (Fig. 9B).

## Discussion

WRKY TFs are one of the largest gene families, forming a vital component of plant signal transduction network for modulating multiple stress response processes in plants [28]. In recent years, many WRKY proteins have been identified from a variety of plants such as rice [36], soybean [37], cotton [31], maize [38], and wheat [39]. Relative to the number of WRKYs identified in cotton, knowledge about their function and molecular mechanism is still unclear, although part of the genome has been comprehensively analyzed according to their functions in the model plants [40]. Therefore, characterization and function of WRKYs in cotton will obtain novel insights into the regulatory mechanism mediated by WRKYs under stress conditions. Here, except for the identification of the WRKY TF *GhWRKY70* from the transcriptome and verification of its function in resistance to Verticillium wilt, we further demonstrated that GhWRKY70 is a positive regulator of *GhAOS1* expression, a key enzyme in the JA biosynthetic pathway. Thus, our study reveals a new mechanism of *GhWRKY70* and links the function of WRKY to JA biosynthesis.

Each WRKY TF possesses two or one domains composed of 60 amino acids with a highly conserved WRKYGQK motif, and there is Cys2His2 or Cys2HisCys zinc-finger motif behind the WRKYGQK motif [28]. GhWRKY70 was classified into the Group III category according to its conserved WRKYGQK domain and zinc finger structure (Fig. 1D). It has been previously demonstrated that most Group III TFs of plants are involved in different plant defense signaling pathways [29, 41–43]. In this study, the results from gene relationship analysis, subcellular localization and transcriptional activation analysis (Figs. 1, 3 and 4) were in accordance with its putative role as a transcription factor. Overexpression of *GhWRKY70* conferred enhanced *Arabidopsis* resistance to *V. dahliae*, suggesting that *GhWRKY70* may act as a positive regulator in response to *V. dahliae*. Taken together, previous studies and our findings indicated that WRKY group III genes play significant and complex roles in the defense against biotic stresses.

Since the expression of *GhWRKY70* was obviously induced by *V. dahliae* and MeJA treatment, we attempted to confirm its role in response to *V. dahliae* by obtaining transgenic *Arabidopsis* with *GhWRKY70*. Here, the transgenic lines overexpressing *GhWRKY70* exhibited better root, height, fresh weight, and chlorophyll and JA contents than WT under treatment with *V. dahliae*, indicating that overexpression of *GhWRKY70* significantly enhanced *V. dahliae* resistance by mediating the JA signaling pathway. Consistent with previous studies, *GhWRKY70A05a* acted as cotton’s resistance against *V. dahliae* by inhibiting the JA signaling pathway, while promoting the SA signaling pathway [34]. *GhWRKY70D13* negatively regulates cotton’s response to *V.dahliae* infection by down-regulating the ET and JA signaling pathways, a mechanism different from that of *GhWRKY70A05a* [35]. However, our study showed that down-regulation of *GhWRKY70* in cotton significantly reduced the accumulation of JA, suggesting that each WRKY plays a different role and might hold great potential for stress tolerance.

A previous study showed that H_2_O_2_ accumulation is closely related to biotic or abiotic stress. Plant cells depend greatly on the antioxidant defense system to maintain the relative balance of H_2_O_2_, such as SOD, CAT and POD [44]. Research has illustrated that H_2_O_2_ can diffuse across membranes and act as a signal during cell wall synthesis and fortification associated with disease resistance [15, 45]. The present study indicated that the content of H_2_O_2_ in *TRV:GhWRKY70* cotton was relatively lower than that in *TRV:00* cotton under *V. dahliae* infection. Further work showed that SOD activity decreased in *TRV:00* plants, *TRV:GhWRKY70* plants, WT and overexpression *Arabidopsis* plants after *V. dahliae* infection. The results indicated that *V. dahliae* can destroy the homeostasis of the active oxygen metabolism system in plants. However, the SOD activity of WT *Arabidopsis* and *TRV:GhWRKY70* decreased rapidly, while the SOD activity of transgenic *Arabidopsis* and *TRV:00* plants decreased more gently, indicating that the latter plants have resistance to *V. dahliae* to a certain extent. The CAT activity of the overexpression plants decreased significantly compared with that of the WT, while the CAT activity of *TRV:GhWRKY70* increased slightly more than that of *TRV:00* after treatment with *V. dahliae*. This may be one of the reasons why the content of H_2_O_2_ in transgenic *Arabidopsis* is higher than that in WT, while the opposite was true for gene silenced plants. Studies have shown that CAT expression also relates to JA [46]. Overexpressing CATALASE2 increases plant JA content and resistance to *Botrytis cinerea* B05.10 infection [47]. In addition, MYC2 could directly bind to the promoter of CAT2 and inhibit its expression [48], which may be one of the reasons for increased H_2_O_2_ in overexpressed plants. Therefore, we speculated that H_2_O_2_ may be one of the key downstream factors of JA signaling pathway in the immune reaction against *V. dahliae*.

PAL and PPO have been confirmed to be involved in plant resistance to fungal infection and can be used as innate immunity markers in plants [49–51]. PAL is a key enzyme in lignin synthesis. Compared with wild type tobacoo, transgenic tobacco overexpressing of the PAL gene showed high resistance to necrotrophic pathogens [52]. After melon was infected with powdery mildew (*Podosphaera fusca*), resistant varieties could accumulate more lignin than susceptible varieties, and lignin accumulation was positively correlated with PAL expression levels [53]. PPO can not only promote the synthesis of quinine by catalyzing the oxidation of phenolic compounds, but also produce pre-benzoic acid, which is the precursor of lignin synthesis [50, 51]. The expression of the PPO gene in resistant varieties of olives was significantly higher than that in sensitive varieties after *V. dahliae* inoculation, and the contents of phenolic compounds and lignin were also higher than those in susceptible varieties [54]. In our study, the increase in defensive enzyme activities was significantly induced and inhibited in *GhWRKY70* overexpressing and *GhWRKY70*-silenced plants, respectively, following *V. dahliae* inoculation (Fig. 7). The results suggest that *GhWRKY70* may promote lignin synthesis by increasing PAL and PPO enzyme activities, which further confirmed that *GhWRKY70* might act as a positive regulator in resistance to *V. dahliae*.

To further explore the function of *GhWRKY70* in defense against *V. dahliae*, the relative expression levels of the JA biosynthesis genes *LOX* and *AOS* and the JA signal response genes *JAZ3* and *MYC2* were monitored before and after *V. dahliae* treatment in overexpressed and gene-silenced plants. Contrary to gene-silenced plants, the expression of* LOX* and *AOS* was enhanced in transgenic *Arabidopsis* after *V. dahliae* infection. The results were consistent with the changes in JA content in the overexpression and gene-silenced plants. We speculated that *GhWRKY70* may be related to JA synthesis. In this study, a W-Box element exists in the *GhAOS1* promoter, and the interaction between GhWRKY70 and the *GhAOS1* promoter was further verified by transient expression assays. These data suggested that *GhAOS1* is a target gene of GhWRKY70. JAZ3, as a negative regulator and early response gene of the JA signaling pathway, is ubiquitinated and degraded when JA content is high [6, 55]. *MYC2*, as the target gene of JAZs protein, is released and activates the expression of JA signaling pathway downstream related genes accompanying JAZs protein degradation. After infection with *V. dahliae*, a high level of JA is rapidly formed in overexpressed *Arabidopsis*, JAZ protein is degraded, and MYC2 is released, which maintains the continuous opening of the JA signaling pathway and enhances the disease resistance of the plant. However, the expression of JAZ3 increased and MYC2 declined in silenced plants, and then the JA signaling pathway was closed, which reduced the resistance to *V. dahliae*.

## Conclusion

Our work suggested that *GhWRKY70* plays the role of a positive regulator in defenses against *V. dahliae*, which may be partly attributed to its role in influencing JA biosynthesis by regulating *GhAOS1* expression. Invasion of *V. dahliae* promotes *GhWRKY70* expression, which further regulates *GhAOS1* expression by interacting with the W-box of the *GhAOS1* promoter. Up-regulation of *GhAOS1* enhances JA content and further activates the response gene of the JA signaling pathway, accompanied by H_2_O_2_ generation and scavenging, improving its ability to resist *V. dahliae*. These results indicate that *GhWRKY70* is involved in a complex signal regulatory network in response to *V. dahliae*. This work provides novel insight into the molecular mechanisms of JA synthesis and H_2_O_2_ relative homeostasis in resistance to infection with *V. dahliae*. In the future, more work is needed to detect other components in connection with *GhWRKY70* to obtain a clearer picture of the molecular mechanisms by which *GhWRKY70* functions in resistance to *V. dahliae*.

## Methods

### Plant materials and stress treatments

*G. hirsutum* cv. Zhongzhimian 12 (*V. dahliae*-resistant cultivar) and *Arabidopsis thaliana* (ecotype Columbia-0) were obtained from State Key Laboratory of North China Crop Improvement and Regulation, Hebei Agricultural University, Baoding, China. Plants were cultured in a greenhouse with a 16:8 light:dark cycle at 25 °C. All plants were watered weekly with Hoagland’s nutrient solution.

Two-leaf stage cotton seedlings from the greenhouse were treated with either 100 µmol L^− 1^ MeJA or 15% (m/v) polyethylene glycol (PEG–6000). Moreover, for cold stress, the seedlings were moved to a growth chamber set at 4 °C. Cotton leaves were harvested at 0, 2, 6, 12, 24, and 48 h after treatment. For each treatment, at least three randomly collected seedlings at each designated time point were flash frozen in liquid nitrogen and stored at − 80 °C until use [34].

A highly aggressive *V. dahliae* strain linxi2-1, from the State Key Laboratory of North China Crop Improvement and Regulation was cultured on potato dextrose agar (PDA) medium at 25 °C for 7–10 days. Colonies were then moved to Czapek medium by shaking culture (150 r min^− 1^) for 10 days at 25 °C. The culture solution was diluted to 10^7^ conidia mL^− 1^ spore suspensions and then watered (10 mL per treatment) into the pots with soil containing cotton seedlings and *Arabidopsis* plants [56]. The roots of cotton and *Arabidopsis* were cut at 0, 6, 12, 24, and 48 h after infection with *V. dahliae*, flash frozen in liquid nitrogen, and stored at − 80 °C for transcription analysis.

### Isolation and bioinformatics analysis of GhWRKY70

Analysis of the transcriptome data of Zhongzhimian 12 showed that in the seedling growth period, about the seedling grew for approximately two weeks, and were infected with *V. dahliae* and noninfected (normal condition) at 0, 2, 6, 12, 24 and 48 h. For transcriptome analysis, the seedlings with the same growth were cut roots to 1.0 g, with three repeats. RNA library construction, assessment, and all sequencing was performed using Majorbio of Shanghai with HiSeq 2500. The WRKY TF (Ghir_D02G000360) with high sequence homology to GhWRKY70D02 [35] was found to be the most up-regulated in the transcriptome and qRT‒PCR analysis of cotton treated with *V. dahliae* at 2 h. A pair of gene-specific primers (GSP1, Table S2) was designed according to the sequence for amplification of *GhWRKY70* in Zhongzhimian 12. Total RNA was isolated and a cDNA template was prepared. PCR products were purified, ligated into the pMDT-19 vector and sequenced by Sangon Biotech (Shanghai) Co., Ltd. (Shanghai, China). Multiple sequence alignments were performed using Clustal W [57], and a phylogenetic tree was constructed with MEGA 7.0 using the neighbor-joining method [58]. Approximately 2.0 kb of DNA sequence upstream from the codons of *GhWRKY70* was downloaded from *Gossypium hirsutum* HAU genome data in COTTONfgd database (https://cottonfgd.net) [59]. PlantCARE (http://bioinformatics.psb.ugent.be/webtools/plantcare/html/) was used to analyze the promoter region of *GhWRKY70*.

### RNA isolation and expression pattern analysis

Total RNA was extracted from *Arabidopsis* and cotton roots or leaves using a RN09-EASYspin RNA Plant Mini Kit (Aidlab Biotechnologies Co. Ltd., Beijing, China), and digested with DNase I (TaKaRa Biotechnology (Dalian) Co., Ltd., Dalian, China) to eliminate DNA contamination. Approximately 2 µg of total RNA was reverse transcribed into first-strand cDNA using the PrimeScript II 1st Strand cDNA Synthesis Kit (TaKaRa Biotechnology (Dalian) Co., Ltd.). The control *GhUBQ7* (XM_016855110.2) or *AtACTIN2* (AT3G18780), genes involved in the JA signaling pathway, were analyzed by qRT‒PCR with SYBR Green Mix (Takara) and Roche LightCycler 2.0 (Roche, Germany). The primer pairs used for qRT‒PCR analysis were designed with Primer 5 (Table S2). Expression data obtained from three replicate experiments were analyzed by the 2^−ΔΔCt^ method [60] and presented as the means ± SD.

### Subcellular localization of GhWRKY70

The complete ORF sequence of *GhWRKY70* without a stop codon was amplified by RT‒PCR using specific primers (GSP2, Table S2) and then ligated into the pSuper1300-GFP vector using *Hin*dIII and *Sal*I to obtain the fusion protein GhWRKY70::GFP. The nuclear signal peptide (MDPKKKRKV) was fused to the far-RFP mKate of the pBWA(V)HS vector to obtain the pBWA(V)HS-nls-mKate-RFP vector [61]. Both pSuper1300-GhWRKY70-GFP and pBWA(V)HS-nls-mKate-RFP were cotransformed into tobacco leaves. The empty vector pSuper1300-GFP was used as a control. After transformation for 48–72 h, the GFP emission signal was collected at an excitation wavelength of 488 nm under a confocal microscope (Leica), and the RFP signal was observed between 587 and 610 nm.

### Analysis of transcriptional activation in yeast

The full-length coding sequence of *GhWRKY70* was amplified using primers (GSP3, Table S2) with *Eco*RI and *Sal*I restriction sites and fused to the pGBKT7 vector to construct pGBKT7-GhWRKY70. The empty vector pGBKT7 was used as a control. The construction and empty vector pGBKT7 were introduced into the yeast strain AH109 separately by the lithium acetate method. Yeast cells were cultured on selective medium without tryptophan (SD-T). The positive clones identified by PCR were cultured on the SD-T, SD without tryptophan, histidine, and adenine (SD-T/H/A) and SD-T/H/A with X-D-galactosidase (X-gal) medium at 30 °C. The results were observed after 3 d to detect transcriptional activation.

### Generation of *GhWRKY70*-overexpressing *Arabidopsis* plants

The ORF of *GhWRKY70* was cloned into the expression vector pCamE carrying the hygromycin B resistance gene after PCR amplification using a primer pair (GSP4, Table S2) with *Bam*HI and *Kpn*I restriction sites. The binary vector pCamE-*GhWRKY70* was introduced into *Agrobacterium tumefaciens* strain GV3101 and then transformed into WT *Arabidopsis* plants using the floral-dip method [62].

### VIGS and pathogen inoculation

Tobacco rattle virus (TRV)-based vectors and *A. tumefaciens* were used for VIGS [63]. *TRV:GhCLAI* (*Cloroplastos Alterados* 1) was selected as a positive control [64]. A fragment of *GhWRKY70* was digested with *Bam*HI and *Kpn*I and then cloned into the *TRV:00* plasmid to construct the VIGS vector of *TRV:GhWRKY70*. Silencing sequence of *GhWRKY70* and *GhCLAI* (NM_001327127.1) is shown in Fig S4. The constructs were introduced into *A. tumefaciens* GV3101 as described previously [3, 65]. A *TRV1* (helper) and *TRV:GhWRKY70 Agrobacterium* (OD600 = 1.0) mixture (1:1 ratio, v/v) was agroinfiltrated into the cotyledons of 7-day-old cotton seedlings. These treated seedlings were cultured for 12 h in darkness and then grown in a controlled environment greenhouse. Leaves of the *TRV:GhWRKY70* and the *TRV:00* plants were picked for RNA isolation after the *TRV:GbCLA1* plants were observed to have a leaf bleaching phenotype. Some *TRV:GhWRKY70* and *TRV:00* plants were infected with *V. dahliae* to observe phenotypic traits and to score the disease index (DI). The plant DI was calculated according to the method described by Xu et al. [66].

### Analysis of antioxidant enzyme and defense enzyme activities

For SOD, POD and CAT activity detection, fresh tissue (0.4 g) was homogenized with 0.1 M phosphate-buffered saline (PBS) (5 mL, pH 7.5). Then, the homogenate was centrifuged at 12,000×g at 4 °C for 15 min, and the supernatant was collected for enzymatic detection [67]. SOD and POD activities were analyzed according to Giannopolitis and Ries [68] and Doerge et al. [69], respectively. CAT activity was measured by monitoring the consumption of hydrogen peroxide (H_2_O_2_) at 240 nm [70]. The enzyme activities were expressed as Ug^− 1^ fresh weight (FW). To determine PAL and PPO enzyme activities, fresh tissue (0.4 g) was ground using 4 mL of 0.2 mM boric acid buffer (pH 8.8) containing 10% (w/v) PVP, 5 mM β-mercaptoethanol, 1 mM EDTA and 0.1 M of sodium phosphate buffer (pH 7.8) containing 1% (w/v) PVP. The homogenates were centrifuged at 12,000×g for 15 min at 4 °C, and the supernatants were used for enzyme assays [71].

### 3, 3′-diaminobenzidine (DAB) staining and lignin histochemical staining

For H_2_O_2_ determination, leaves were soaked in 1 mg/mL pH 3.8 DAB-HCl (Sigma‒Aldrich, USA) for 8 h in the dark, and then cleared by boiling in 95% ethanol for 10 min. The reddish color of the leaves served as a visual marker of H_2_O_2_ production. After *V. dahliae* inoculation, the stem base of the plants was taken for transverse sectioning. The free-hand sections (approximately 0.5 mm thick) were soaked in lignin acidification solution for 5 min. Then, the same amount of phloroglucinol staining solution was added to the transverse section and immersed for 10 min. The lignin staining results were observed under an optical microscope.

### Isolation of the *GhAOS1* promoter and Y1H assay

The promoter sequence of *GhAOS1* was acquired by RT–PCR using special primer (GSP5, Table S2). Potential cis-acting elements related to stress resistance were predicted by PlantCARE. Based on the characteristics of the promoter sequence, the fragment (–854 bp to –683 bp) containing potential cis-acting elements W-box with *Hin*dIII and *Xho*I restriction sites was chemically synthesized and integrated into pAbAi to generate the pAbAi-GhAOS1 reporter vector. Then, the *GhWRKY70* full-length ORF with the stop codon removed was amplified using GSP6 (Table S2) and ligated into the *Eco*I and *Xho*I sites of pGADT7 to obtain the pGADT7-GhWRKY70 effector vector. Following the instructions of the Matchmaker Gold Y1H Library Screening System (Clontech, Dalian, China), the interaction of GhWRKY70 and the *GhAOS1* promoter was examined by yeast one-hybrid assays. pAbAi-GhAOS1 and pGADT7-GhWRKY70 were cotransformed into yeast cells. The transformed yeast cells were cultured on SD/-Ura/-Leu medium either with or without 200 ng/mL aureobasidin A (AbA) for 3 days.

### Transient expression assay

The coding region of *GhWRKY70* was amplified using primers (GSP7, Table S2) containing the *Bam*HI and *Eco*RI restriction sites and inserted into pGreenII 62-SK digested by the same enzymes. A 150-bp promoter fragment of *GhAOS1* with a W-box element (P2) and its mutated sequence (mP2) were chemically synthesized containing either *Pst* I or *Bam*H I restriction sites and ligated into the reporter vector, pGreen II 0800-LUC [72]. The reporter and effector constructs were transformed into *A. tumefaciens* GV3101 cells. Assays for transient expression in tobacco leaves were performed as described previously [73]. The transformed tobacco was placed in the dark at 25 °C for 18 h, and then the activity of Renilla luciferase (REN) and firefly luciferase (LUC) was analyzed by the Dual-Luciferase-Reporter Assay System (Promega, Madison, WI, USA) with an Infinite200 Pro reader (Tecan, M€;annedorf, Switzerland). The promoter activity was described as the ratio of LUC/REN.

### Statistical analysis

All data were expressed as the means ± standard deviations (SD) and compared by two-group *t test* comparisons at *P* < 0.05 or *P* < 0.01 using Origin software 8.5.

## Electronic supplementary material

Below is the link to the electronic supplementary material.

Supplementary Material 1

Supplementary Material 2

## Data Availability

All data generated or analyzed during this study are included in this published article and its supplementary information files; Sequence data from this article can be found in the TAIR/GenBank data libraries under the following accession numbers: AtLOX1, AT1G55020; AtAOS, AT5G42650; AtJAZ3, AT3G17860; AtMYC2, AT1G32640; AtACTIN2, AT3G18780; GhCLAI, NM_001327127.1; GhLOX1, XM_041108768.1; GhAOS1, XM_016842008.2; GhJAZ3, XM_041107142.1; GhMYC2, XM_016865820.2; GhUBQ7, XM_016855110.2.

## Abbreviations

JA: Jasmonic acid
MeJA: Jasmonic acid methyl ester
PEG: Polyethylene glycol
SA: Salicylic acid
ROS: Reactive oxygen species
H_2_O_2_: Hydrogen peroxide
SOD: Superoxide dismutase
POD: Peroxidase
CAT: Catalase
PAL: Phenylalanine ammonialyase
PPO: Polyphenol oxidase
VIGS: Virus-induced gene silencing
qRT-PCR: Real-time quantitative PCR
DAB:3: 3′-diaminobenzidine
