# Supplementary material for: The transcription factor GhWRKY70 from gossypium hirsutum enhances resistance to verticillium wilt via the jasmonic acid pathway
Source: BMC Plant Biol. 2023 Mar 14;23:141. doi: 10.1186/s12870-023-04141-x (PMC10012446; doi:10.1186/s12870-023-04141-x)
Supplement: Supplementary file 1 — Supplementary Material 1 [file 12870_2023_4141_MOESM1_ESM.docx]

Table S1 Accession numbers of 72 WRKYs from *Arabidopsis* in TAIR data libraries

| Gene name | accession numbers | Gene name | accession numbers |
| --- | --- | --- | --- |
| AtWRKY1 | AT2G04880 | AtWRKY40 | AT1G80840 |
| AtWRKY2 | AT5G56270 | AtWRKY41 | AT4G11070 |
| AtWRKY3 | AT2G03340 | AtWRKY42 | AT4G04450 |
| AtWRKY4 | AT1G13960 | AtWRKY43 | AT2G46130 |
| AtWRKY6 | AT1G62300 | AtWRKY44 | AT2G46130 |
| AtWRKY7 | AT4G24240 | AtWRKY45 | AT3G01970 |
| AtWRKY8 | AT5G46350 | AtWRKY46 | AT2G46400 |
| AtWRKY9 | AT1G68150 | AtWRKY47 | AT4G01720 |
| AtWRKY10 | AT1G55600 | AtWRKY48 | AT5G49520 |
| AtWRKY11 | AT4G31550 | AtWRKY49 | AT5G43290 |
| AtWRKY12 | AT2G44745 | AtWRKY50 | AT5G26170 |
| AtWRKY13 | AT4G39410 | AtWRKY51 | AT5G64810 |
| AtWRKY14 | AT1G30650 | AtWRKY52 | AT5G45260 |
| AtWRKY15 | AT2G23320 | AtWRKY53 | AT4G23810 |
| AtWRKY16 | AT5G45050 | AtWRKY54 | AT2G40750 |
| AtWRKY17 | AT2G24570 | AtWRKY55 | AT2G40740 |
| AtWRKY18 | AT4G31800 | AtWRKY56 | AT1G64000 |
| AtWRKY19 | AT4G12020 | AtWRKY57 | AT1G69310 |
| AtWRKY20 | AT4G26640 | AtWRKY58 | AT3G01080 |
| AtWRKY21 | AT2G30590 | AtWRKY59 | AT2G21900 |
| AtWRKY22 | AT4G01250 | AtWRKY60 | AT2G25000 |
| AtWRKY23 | AT2G47260 | AtWRKY61 | AT1G18860 |
| AtWRKY24 | AT5G41570 | AtWRKY62 | AT5G01900 |
| AtWRKY25 | AT2G30250 | AtWRKY63 | AT1G66600 |
| AtWRKY26 | AT5G07100 | AtWRKY64 | AT1G66560 |
| AtWRKY27 | AT5G52830 | AtWRKY65 | AT1G29280 |
| AtWRKY28 | AT4G18170 | AtWRKY66 | AT1G80590 |
| AtWRKY29 | AT4G23550 | AtWRKY67 | AT1G66550 |
| AtWRKY30 | AT5G24110 | AtWRKY68 | AT3G62340 |
| AtWRKY31 | AT4G22070 | AtWRKY69 | AT3G58710 |
| AtWRKY32 | AT4G30935 | AtWRKY70 | AT3G56400 |
| AtWRKY33 | AT2G38470 | AtWRKY71 | AT1G29860 |
| AtWRKY34 | AT4G26440 | AtWRKY72 | AT5G15130 |
| AtWRKY35 | AT2G34830 | AtWRKY74 | AT5G28650 |
| AtWRKY36 | AT1G69810 | AtWRKY75 | AT5G13080 |
| AtWRKY38 | AT5G22570 |  |  |
| AtWRKY39 | AT3G04670 |  |  |

Table S2 Primers used for qRT‒PCR and RT‒PCR

| Primers or genes | Sequences |
| --- | --- |
| GSP1 | 5'-ATGTCTTGGAATACAAAAAAAGCAATCG |
|  | 5'-TCAAAACTGAAACTGAAGCAAATCATCA |
| GSP2 | 5'- CAAATCGACAAGCTTATGTCTTGGAATACAAAAAAAGCAATCG |
|  | 5'-CCACTAGTATTTAAATGGTCGACAAACTGAAGCAAATCATCAAGATGTAC |
| GSP3 | 5'-ATATGGCCATGGAGGCCGAATTCATGTCTTGGAATACAAAAAAAGCAATCG |
|  | 5'- TTATGCGGCCGCTGCAGGTCGACTCAAAACTGAAGCAAATCATCAAGA |
| GSP4 | 5'-CGCGGATCCATGTCTTGGAATACAAAA AAAGCAATCG |
|  | 5'- CGGGGTACCTCAAAACTGAAGCAAATCATCAAGA |
| GSP5 | 5'- CAAATTGTCACTTGGAGCTCGCTGGC |
|  | 5'- TAGGCACGTGGACGGTTTGCAGTAACG |
| GSP6 | 5'-TGGCCATGGAGGCCAGTGAATTCATGTCTTGGAATACAAAAAAAGCAATCG |
|  | 5'-CTACGATTCATCTGCAGCTCGAGTCAAAACTGAAGCAAATCATCAAGA |
| GSP7 | 5'-TGGCCATGGAGCTCCGGATCCATGTCTTGGAATACAAAAAAAGCAATCG |
|  | 5'-CTACGATTCATCTGCAGGAATTCTCAAAACTGAAGCAAATCATCAAGA |
| *GhUBQ7* | 5'-GAAGGCATTCCACCTGACCAAC |
|  | 5'-CTTGACCTTCTTCTTCTTGTGCTTG |
| *GhWRKY70* | 5'-GCCGTCGCCGATTATTAT |
|  | 5'- TGCGTAGAGGGAGGACTTT |
| *GhLOX1* | 5'- GCTTATGTTGCTGTAAATGACTCTGG |
|  | 5'-CACACTAAGTTGTCGGTTCGTTG |
| *GhAOS* | 5'-TGCCACCTGGTCCTTTCATTTC |
|  | 5'-GCGTGTTTGGGCTCGGAAGGGTCG |
| *GhJAZ3* | 5'-TGATTTTGCTCAAGGAGATAACGCT |
|  | 5'-TGATTGCCTACTCGTTGCCTGT |
| *GhMYC2* | 5'-GCTCCGCCACTACCGTGCTC |
|  | 5'-CTCGAAGCACTTTTTTACGGTGTTC |
| *AtACTIN2* | 5'-ACAGTGTCTGGATCGGTGGTTC |
|  | 5'- TGCCTCATCATACTCAGCCTTG |
| *AtLOX1* | 5'-GTGGATGGGCTTGAGGTTTGGTAT |
|  | 5'-TTCTTCACGGGTTTGCATTTTAGG |
| *AtAOS* | 5'-AACACCAGCTCCAGCTCTATTCTT |
|  | 5'-TTGACTCTGTACACCGTGGAGTT |
| *AtJAZ3* | 5'-TATCCCCATATTGCTTAGAC |
|  | 5'-CAAATATGGATGTGGAGTG |
| *AtMYC2* | 5'-GTCGGCGTTGATGGATTTGGAGT |
|  | 5'-CTTGCTCTGAGCTGTTCTTGAGTATAGATC |
