# Supplementary material for: The transcription factor GhWRKY70 from gossypium hirsutum enhances resistance to verticillium wilt via the jasmonic acid pathway
Source: BMC Plant Biol. 2023 Mar 14;23:141. doi: 10.1186/s12870-023-04141-x (PMC10012446; doi:10.1186/s12870-023-04141-x)
Supplement: Supplementary file 2 — Supplementary Material 2 [file 12870_2023_4141_MOESM2_ESM.docx]

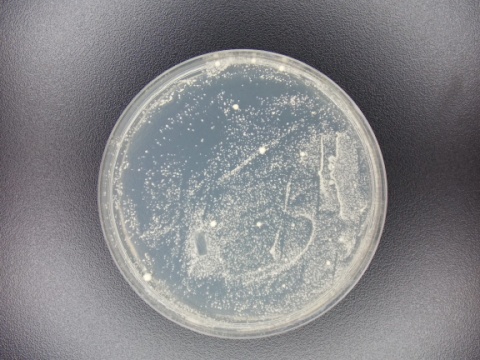

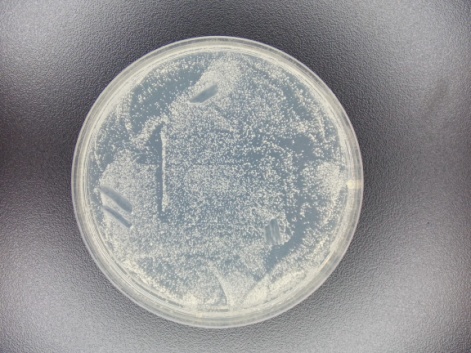

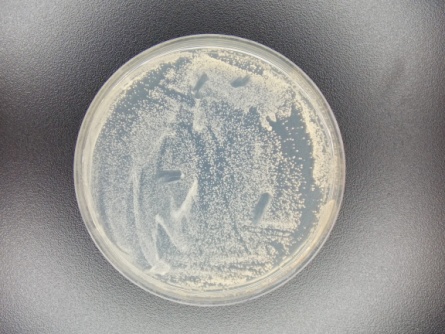

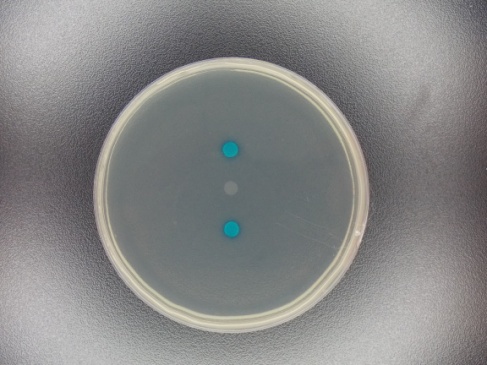

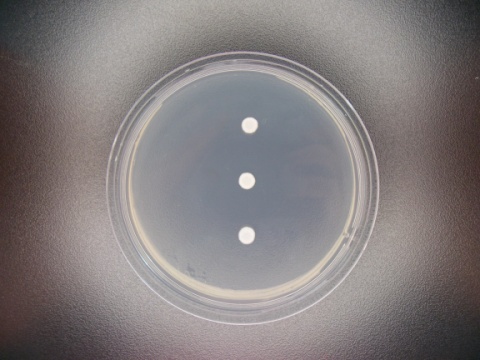

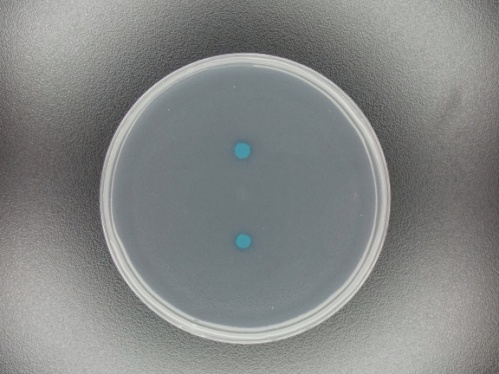


Y2Hgold[pGBKT7] Y2Hgold[pGBKT7-GAL4] Y2Hgold[pGBKT7-GhWRKY70]

SD-Trp SD-Trp-His SD-Trp-His-Ade

Fig. S1 Transcriptional activation assay of GhWRKY70 in yeast


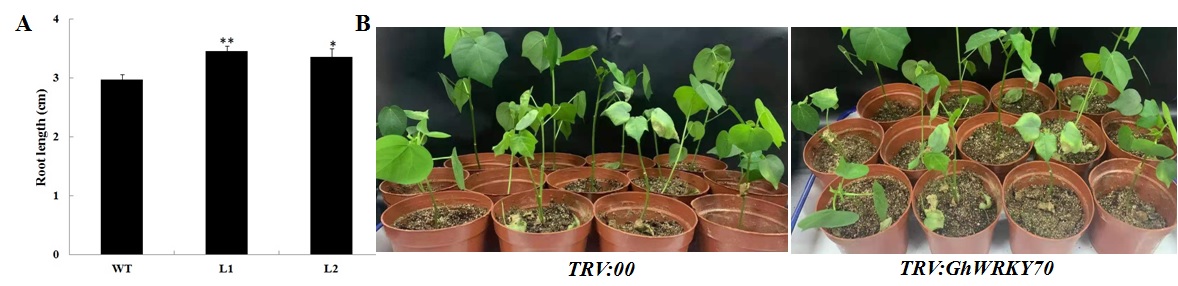
Fig.S2 The root length of WT and transgenic *Arabidopsis* and VIGS of *GhWRKY70* in cotton. (A) Root length of transgenic and WT *Arabidopsis* on 9 d of growth. (B) Disease symptoms induced in empty-vector control (*TRV:00*) and *TRV:GhWRKY70* cotton plants after *V. dahliae* inoculation 15 days.


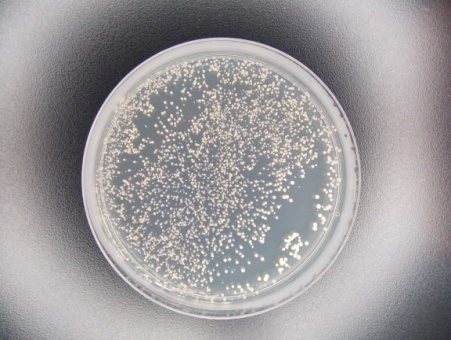

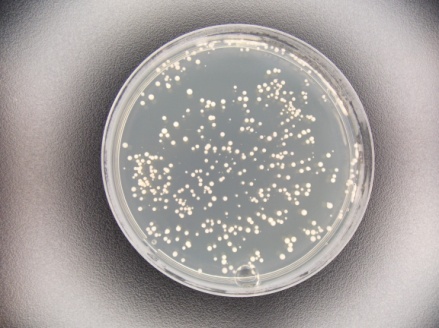

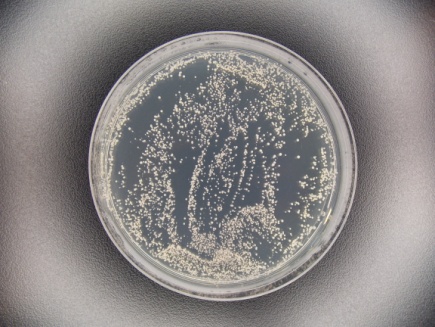


Y1Hgold[p53-AbAi&pGAD-Rec-p53] Y1Hgold[pG-box- AbAi&pGADT7-GhWRKY70] Y1Hgold[pG-boxmutant- AbAi&pGADT7-GhWRKY70]


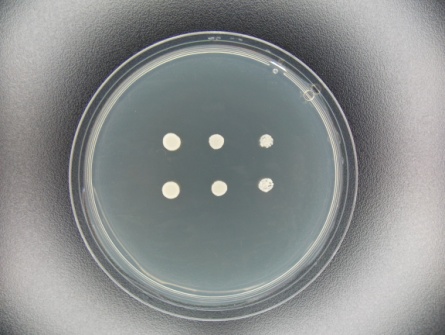

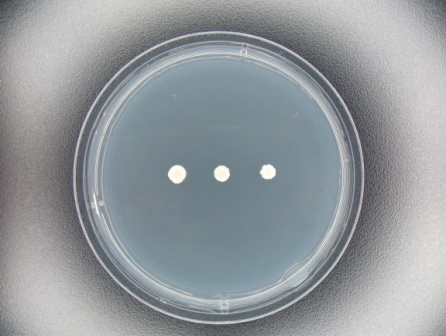


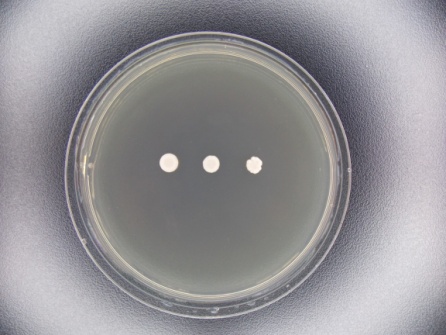


SD/-Ura/-Leu SD-Ura/-Leu SD-Ura/-Leu


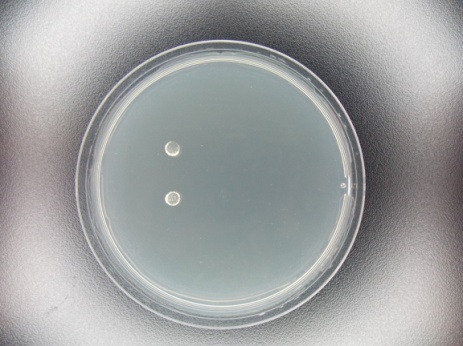

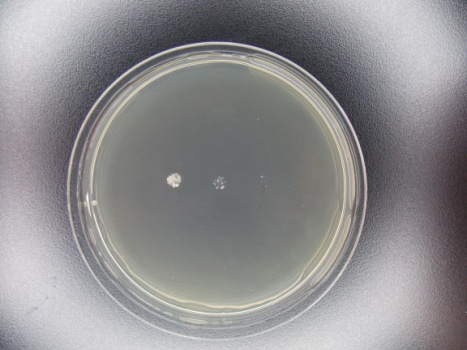

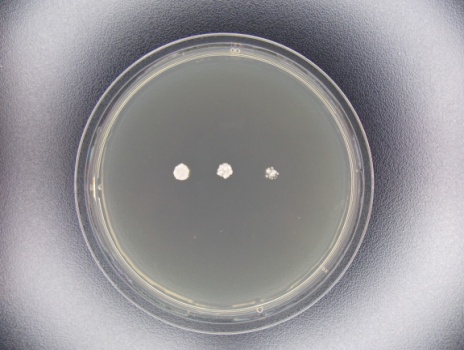


SD/-Ura/-Leu+200ngAba SD/-Ura/-Leu+200ngAba SD/-Ura/-Leu+200ngAba

Fig. S3 Yeast one-hybrid (Y1H) assay between GhWRKY70 and the GhAOS1 promoter


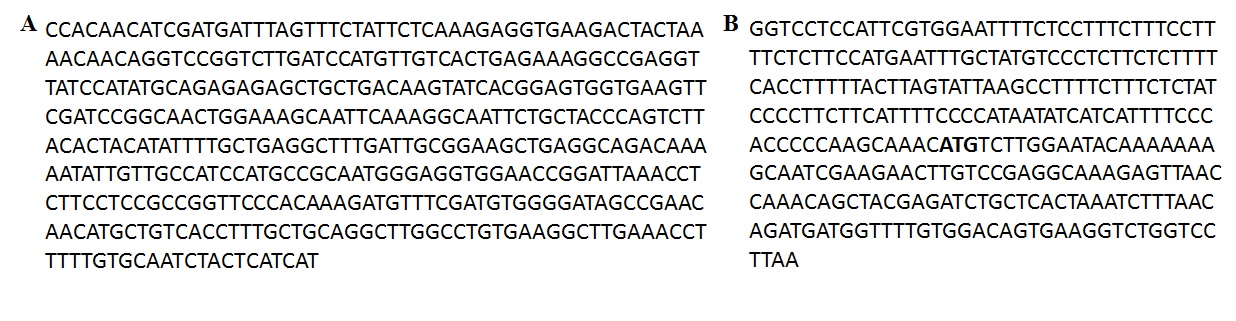


Fig. S4 Silencing sequence of GhCLA1 (422bp) (A) and GhWRKY70 (296bp) (B) for VIGS.
